# MET phosphorylation predicts poor outcome in small cell lung carcinoma and its inhibition blocks HGF-induced effects in MET mutant cell lines

**DOI:** 10.1038/bjc.2011.298

**Published:** 2011-08-16

**Authors:** E Arriola, I Cañadas, M Arumí-Uría, M Dómine, J A Lopez-Vilariño, O Arpí, M Salido, S Menéndez, E Grande, F R Hirsch, S Serrano, B Bellosillo, F Rojo, A Rovira, J Albanell

**Affiliations:** 1 Oncology Department, Hospital del Mar-Parc de Salut Mar, Passeig Marítim 25-29, 08003, Barcelona, Spain; 2 Cancer Research Program, IMIM-Hospital del Mar, Dr Aiguader, 88, 08003, Barcelona, Spain; 3 Pathology Department, Hospital del Mar-Parc de Salut Mar, Passeig Marítim 25-29, 08003, Barcelona, Spain; 4 Oncology Department, IIS-Fundación Jiménez Díaz, Avenida Reyes Católicos 2, 28040, Madrid, Spain; 5 Department of Health and Experimental Sciences, Universitat Pompeu Fabra, Plaça de la Mercè, 10-12, 08002, Barcelona, Spain; 6 Oncology Department, Hospital Ramón y Cajal, Madrid, Spain; 7 University of Colorado Cancer Center, Aurora, CO, USA; 8 Pathology Department, IIS-Fundación Jiménez Díaz, Avenida Reyes Católicos 2, 28040, Ctra. de Colmenar Viejo km. 9,100 28034, Madrid, Spain; 9 Autonomous University of Barcelona, 08193 Bellaterra, Cerdanyola del Vallès, Spain

**Keywords:** small cell lung cancer, HGF, MET, H69, PHA-665752, mutation

## Abstract

**Background:**

Small cell lung carcinoma (SCLC) has poor prognosis and remains orphan from targeted therapy. MET is activated in several tumour types and may be a promising therapeutic target.

**Methods:**

To evaluate the role of MET in SCLC, *MET* gene status and protein expression were evaluated in a panel of SCLC cell lines. The MET inhibitor PHA-665752 was used to study effects of pathway inhibition in basal and hepatocyte growth factor (HGF)-stimulated conditions. Immunohistochemistry for MET and p-MET was performed in human SCLC samples and association with outcome was assessed.

**Results:**

In MET mutant SCLC cells, HGF induced MET phosphorylation, increased proliferation, invasiveness and clonogenic growth. PHA-665752 blocked MET phosphorylation and counteracted HGF-induced effects. In clinical samples, total MET and p-MET overexpression were detected in 54% and 43% SCLC tumours (*n*=77), respectively. MET phosphorylation was associated with poor median overall survival (132 days) *vs* p-MET negative cases (287 days)(*P*<0.001). Phospho-MET retained its prognostic value in a multivariate analysis.

**Conclusions:**

MET activation resulted in a more aggressive phenotype in MET mutant SCLC cells and its inhibition by PHA-665752 reversed this phenotype. In patients with SCLC, MET activation was associated with worse prognosis, suggesting a role in the adverse clinical behaviour in this disease.

Small cell lung carcinomas (SCLCs) account for ∼15% of lung cancers (Cooper and Spiro, 2006; Govindan *et al*, 2006). Patients with SCLC show an excellent initial tumour response to chemotherapy, however, the majority have a very poor outcome (Spira and Ettinger, 2004; Cheng *et al*, 2007).

Several studies on genetic changes and potential molecular targets in SCLC have been performed (Arriola *et al*, 2008). Disappointingly, however, the standards of treatment and outcome have remained unchanged for the past decade. One of these drugable targets, the *MET* gene, encodes for a heterodimeric transmembrane receptor tyrosine kinase composed of an extracellular *α*-chain (50 kDa) disulphide bonded to a membrane-spanning *β*- (145 kDa) chain (Park *et al*, 1987; Giordano *et al*, 1989). The hepatocyte growth factor (HGF) is the natural ligand of MET (Naldini *et al*, 1991; Rosen *et al*, 1994). Upon receptor binding HGF induces phosphorylation of several sites on the juxtamembrane and intracellular domains and mediates, through the interaction with Gab1 (Weidner *et al*, 1996; Sachs *et al*, 2000), the activation of several signalling pathways, such as ERK, AKT, PKC*α* and paxillin FAK (Ponzetto *et al*, 1994; Furge *et al*, 2000). This activation results in cellular effects that stimulate invasiveness, tubule formation and branching (Birchmeier *et al*, 2003).

Activating mutations and *MET* gene amplification have been found in lung cancer cell lines and primary tumours, resulting in the constitutive activation of the pathway and its cellular effects in cell line models (Bardelli *et al*, 1998; Peschard *et al*, 2001; Ma *et al*, 2003; Kong-Beltran *et al*, 2006; Engelman *et al*, 2007; Lutterbach *et al*, 2007). Inhibition of MET in these cases has led to inhibition of growth and migration/invasiveness (Christensen *et al*, 2003; Sattler *et al*, 2003; Puri *et al*, 2007; Zou *et al*, 2007; Corso *et al*, 2008). Furthermore, a number of studies have assayed the expression of MET and, to a lesser extent, p-MET (i.e., activated MET) expression in various human cancers. There are increasingly more consistent data to suggest that total MET and in particular p-MET expression show association with poor survival of cancer patients (Furukawa *et al*, 1995; Belfiore *et al*, 1997; Qian *et al*, 2002; Birchmeier *et al*, 2003; Puri *et al*, 2007; Tuynman *et al*, 2008; Benedettini *et al*, 2010). In addition, initial results from two phase II studies in patients with advanced NSCLC have recently been reported and demonstrated the therapeutic value of targeting the MET receptor in combination with EGFR tyrosin kinase inhibitors (Schiller *et al*, 2010; Spigel *et al*, 2010).

Small cell lung carcinoma is characterized by high invasive and metastatic capacity, a typical feature of MET-activated tumour models (Comoglio and Trusolino, 2002; Danilkovitch-Miagkova and Zbar, 2002) and previous studies suggest a role for MET in SCLC (Maulik *et al*, 2002; Jagadeeswaran *et al*, 2007; Ma *et al*, 2007). However, its relevance for SCLC patient outcome and a potential role of MET inhibitors for the treatment of this disease remain to be determined.

To provide further information on MET as a potential target in SCLC, we studied the effects of a MET inhibitor in a panel of SCLC cell lines and also assayed the expression, activation state and mutational status of MET in a series of human SCLC specimens with complete clinical follow up.

## Materials and methods

### Cell lines and reagents

A panel of 10 SCLC cell lines models was screened for MET alterations for this study. The SCLC cell lines H69, H69AR, H187, SHP-77, H345, H740, H748, H865, and H524 and H1688, and the NSCLC H1993 cells were obtained from the American Type Culture Collection (ATCC) (Rockville, MD, USA). All cells were cultured according to the ATCC instructions (http://www.ATCC.org).

Recombinant human HGF was purchased from Calbiochem (La Jolla, CA, USA) and was resuspended in sterile PBS containing 0.1% BSA and stored at a stock concentration of 100 *μ*g ml^−1^ at −80°C. Treatments (unless otherwise indicated) were done at 40 ng ml^−1^ as final concentration in culture medium.

PHA-665752, (3Z)-5-[(2,6-dichlorobenzyl)sulfonyl]-3-[(3,5-dimethyl-4-1H-pyrrol-2-yl)methylene]-1,3-dihydro-2H-indol-2-one (obtained from Pfizer Inc., San Diego, CA, USA) was dissolved to a stock concentration of 10 mM in DMSO at −80°C. The stock solution was diluted for instant use in our experiments.

### Western blot analysis

The following antibodies were purchased from the manufacturers listed below and used for western blot assay: Met (25H2) mouse monoclonal antibody (mAb), p-MET Y1234/1235 (3D7) rabbit mAb, p-MET Y1349 (130H2) rabbit mAb, p-MET Y1234/35 (D26) XP rabbit mAb, p-GAB1 Y307-rabbit polyclonal antibody (pAb), ERK1/2 rabbit pAb, p-ERK1/2 (Thr202/Tyr204) pAb were obtained from Cell Signaling (Danvers, MA, USA) and GAB1 (H198) rabbit pAb from Santa Cruz Biotechnology (Santa Cruz, CA, USA). Western blot from whole-cell extracts was performed as previously reported (Codony-Servat *et al*, 2006).

### Fluorescence *in situ* hybridisation

The status of the *MET* gene in cell lines was assessed by fluorescence *in situ* hybridisation (FISH) using the ON·C-MET (7q31)/SE 7 FISH probes (Kreatech Diagnostics, Amsterdam, The Netherlands), labelling the centromeric alpha-satellite region, specific for chromosome 7 (spectrum green), and the 7q31 region that contains the *MET* gene (spectrum orange), as described (Salido *et al*, 2009).

### Mutational analysis of the *MET* gene by Sanger sequencing

For mutational studies of tumour samples, DNA was extracted from macrodissected tumoural paraffin-embedded tissue using the QIAamp Tissue Kit (QIAGEN GMBH, Hilden, Germany) according to the manufacturer's protocol. The mutational analysis of the *MET* gene in cell lines and tumour samples (codons E168, R988 and T1010) was performed by direct sequencing. Primers for PCR amplification and sequencing were designed using the Primer Express software (Applied Biosystems, Foster City, CA, USA) and were as follows: 5′-GCAGCAGCAAAGCCAATTTAT-3′ and 5′-TGACTTTGGCTCCCAGGGC-3′ for the E168 and 5′-ACCCATGAGTTCTGGGCACT-3′ and 5′-CAGAACAATAAACTGAAATATACCTTCTGG-3′ for the R988 and T1010. PCR conditions were as follows: 95°C 10 min 1 cycle; 95°C 1 min, 55°C for 1 min, 72°C for 1 min, 40 cycles; and 72°C 10 min, 1 cycle. Sequencing was performed with BigDye v3.1 (Applied Biosystems) following the manufacturer's instructions and analysed on a 3500Dx Genetic Analyzer (Applied Biosystems).

### Viability assays

To measure effects of HGF, PHA-665752 or the combination on the viability of SCLC cell lines, we seeded 3 × 10^5^ cells per well in a six-well plate with culture medium containing 10% FBS. After 24 h HGF, PHA-665752 or the combination were added at 40 ng ml^−1^ or 0.5 *μ*M, respectively, and incubated during 72 h. Cell viability was determined by trypan blue/haemocytometer exclusion method. Each experimental condition was done in duplicate. The results were plotted as percentage of control.

### Soft-agar colony formation assay

Single cell suspensions (2 × 10^4^ cells in 35 mm plates) were grown in 0.3% agar containing FBS 10% in RPMI 1640 medium in the presence and absence of HGF (40 ng ml^−1^) and PHA-665752 (0.5 *μ*M) on top of 0.5% agar. The plates were incubated (37°C, 5% CO2) and cells were fed every 3 days with 0.5 ml of culture medium containing the above-mentioned HGF and PHA-665752 concentrations. The number and size of colonies were evaluated at × 20 magnification on a light microscope at each condition after 21 days.

### Invasion assay

To evaluate the invasiveness of SCLC cell lines we prepared cell suspensions in serum-free media and 2.5 × 10^4^ adherent cells or 1 × 10^6^ cells growing in suspension were seeded into the inserts of 24-well (8 *μ*m pore size) CHEMICON Invasion Chamber (CHEMICON International, Inc., Temecula, CA, USA) and treated with PHA-665752 (0.5 *μ*M), a concentration that does not have a significant effect on cell viability. Inserts were placed into Falcon companion plates (BD, San Jose, CA, USA) containing 10% FBS and 40 ng ml^−1^ HGF and incubated for 24 h at 37°C and 5% CO_2_ atmosphere.

Following incubation non-invading cells were removed from the top chamber using cotton swabs and the outside of the insert was gently rinsed with PBS, stained with 0.25% crystal violet for 20 min and rinsed again. Acetic acid (10%, 200 *μ*l per well) was applied to dissolve the stained cells, and then the dye/solute mixture was transferred to a 96-well plate for colorimetric reading of optical density (OD) at 560 nM. The OD value represents the invasive ability. All experiments were performed in triplicate.

### Tumour samples and immunohistochemistry

The following antibodies were used for immunohistochemical studies and purchased from the manufactures listed below: MET (3D4) mouse mAb (Invitrogen, San Francisco, CA, USA), MET (SP44) mouse mAb (Ventana-Roche, Tucson, AZ, USA), p-MET Y1349 (130H2) rabbit mAb, p-MET Y1234/35 (3D7) rabbit mAb, p-MET Y1234/35 (D26) XP rabbit mAb (Cell Signaling) (Benedettini *et al*, 2010) and RON *β* rabbit pAb (C-20) (Santa Cruz Biotechnology). We performed two series of experiments to rule out unspecific staining both in cell lines and tumour samples. First, in both formalin-fixed basal and HGF-treated H69 cell pellets (Supplementary Figure 1), and in human SCLC specimens (Figure 3B), two different anti-MET (3D4 and SP44) antibodies and two anti-p-MET (130H2 and D26) resulted in similar staining patterns in 20 (archival SCLC samples) and 30 specimens (from the current series), respectively. Furthermore, sections from same specimens above were incubated with normal mouse IgG2 (X0943, Dako, Carpinteria, CA, USA) or normal rabbit Ig fraction (X0903, Dako) instead of primary antibodies as negative controls. Second, to rule out potential cross-reactivity with RON (Gaudino *et al*, 1994), we assayed RON, MET and p-MET in a subset of 20 (archival SCLC) tumour specimens by IHC. Specimens RON positive and MET/p-MET negative, and vice versa, were observed (data not shown), thus indicating lack of cross-reactivity in our assays.

The study population consisted of 77 patients diagnosed with SCLC at any stage from whom we had clinical and follow-up information. Biopsies were obtained before treatment and subsequently patients initiated standard chemotherapy regimes. All samples except for two were non-surgical core biopsies as surgery is not a standard procedure in SCLC treatment (usually diagnosed in advanced stages). Specimens were retrospectively retrieved from Parc de Salut Mar Biobank (MARBiobanc, Barcelona, Spain) and Fundación Jiménez Díaz Biobank (Madrid, Spain). This study was approved by the institutional review board of each participating centre. Three-*μ*m tissue sections from formalin-fixed and paraffin-embedded samples were obtained, mounted onto charged slides and then, deparaffinised in xylene and hydrated. After heat antigen retrieval performed at high pH solution using PT Link platform (Dako), slides were incubated with primary antibody for 1 h at a dilution of 1 : 50 for 3D4 MET, 1 : 1 MET SP44 mouse mAb, 1 : 20 for p-MET Y1349 and 1 : 50 for p-MET Y1234/35. Then, sections were incubated with the specific polymer EnVision Flex+ (Dako), revealed with 3–3′ diaminobenzidine, a chromogen, and counterstained with haematoxylin.

Sections were evaluated by two pathologists independently blinded to clinical information on a light microscope (Olympus DX50, Olympus Corp., Tokyo, Japan). The MET and p-MET staining was scored when any percentage of tumour cells was stained in the membrane. A semiquantitative histoscore (Hscore) was calculated, determined by estimation of the percentage of tumour cells positively stained with low, medium, or high staining intensity for each marker. The final score was determined after applying a weighting factor to each estimate. The formula used was Hscore=(low %) × 1+(medium %) × 2+(high %) × 3, and the results ranged from 0 to 300. Staining of non-tumour epithelial cells (normal and metaplastic respiratory bronchial epithelium) was also assessed.

### Statistical analysis

Statistical analysis was carried out with SPSS version 13.0 (SPSS, Inc., Chicago, IL, USA). To analyse correlations between MET status and clinical-pathological variables we used the *χ*^2^-test (Fisher's exact test). Correlations between expression scores by different primary antibodies were calculated by Spearman rho test. Overall survival was analysed by the Kaplan–Meier method. Curves were compared by the log-rank test. Multivariate analysis was performed using the Cox proportional hazards model, including the variables that had reached statistical significance in the univariate analysis. All the statistical tests were conducted at the two-sided 0.05 level of significance. Receiver operating curve (ROC) was used to determine the optimal cutoff point for MET and p-MET overexpression (Baker, 2003). As shown in Results, the cutoff was set for a MET score of 120 and a p-MET Hscore of 5. Specimens with values above these cutoff points were considered as MET or p-MET positive and specimens with values equal or below as negative. Data and statistical analysis reporting are fully compliant with the REMARK guidelines (McShane *et al*, 2005).

## Results

### PHA-665752 inhibits HGF-induced phosphorylation of MET, downstream molecules and HGF-induced proliferation

To characterize MET status in our cell lines, we performed FISH to study the gene copy number, sequencing to study the mutations described on exon 14 for SCLC and protein expression by western blot. None of the 10 SCLC cell lines showed MET amplification. We found *MET* amplification in the NSCLC H1993 cell line as previously reported (data not shown). We therefore used this cell line as a positive control of MET activation. In both H69 and H69AR (chemoresistant) we confirmed the reported juxtamembrane mutation R988C on exon 14 of the *MET* gene (data not shown) (Ma *et al*, 2003; Jagadeeswaran *et al*, 2007). None of the remaining eight SCLC cell lines had R988C or T1010I *MET* mutations on exon 14. We observed total MET expression by WB in H69, H69AR, H187 and H345 SCLC cells. The H865 cell line presented lower levels of MET expression and the remaining cell lines (H524, SHP-77, H748 and UMC-19) showed lack of MET expression. Based on these results we selected H69 as a MET mutant model and H524 (no expression of MET), H187 (MET expression) and H345 (MET expression) as models of wild-type MET cell lines. Results with H69 were confirmed with H69AR (chemoresistant isogenic cell line also harbouring the R988C mutation).

**Figure 1:**
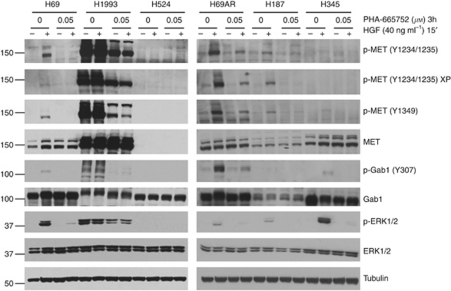
Hepatocyte growth factor activates MET and downstream molecules in mutant SCLC cell line and PHA-665752 inhibits these effects. Cells were serum starved for 24 h and then treated with PHA-665752 (0.05 *μ*M) for 3 h and stimulated with HGF (40 ng/ml) during 15′. Whole cell lysates were purified, size separated by SDS–PAGE, transferred to a membrane and probed with various antibodies.

To further characterize the MET pathway in these selected cells, we performed a western blot analysis of total and phosphorylated MET, and downstream relevant molecules, ERK and GAB-1. As expected, our positive control, the *MET* amplified H1993 cell line, showed high levels of basal and phosphorylated MET and absence of modulation by HGF 40 ng ml^−1^. Figure 1 illustrates the basal and HGF-stimulated expression of these markers in SCLC cells. Basal expression of total MET was detected in H69, H187 and H345 but not in the H524 line. Two bands of 170 and 145k Da were observed corresponding to the unprocessed and mature proteins, respectively. Phosphorylated MET levels were undetectable in H69, H187, H345 and H524 at basal conditions. When stimulated by HGF (15′), p-MET was upregulated in the H69 and H187 cells, and slightly for H345; remaining unchanged for H524. We observed induction of phosphorylation of ERK and GAB-1 with the addition of HGF in H69, H187 and H345 cells. PHA-665752 was able to inhibit MET and phosphorylation of downstream molecules in a dose-dependent manner in H69, H187, H345 and H1993 (Figure 1), obtaining a complete inhibition at 0.5 *μ*M (data not shown).

**Figure 2:**
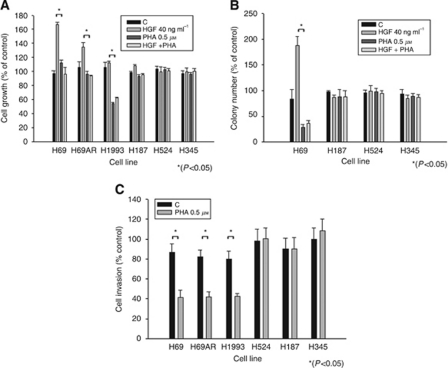
PHA-665752 inhibits HGF-induced cellular effects in MET mutant SCLC cells. (**A**) The PHA-665752 inhibits HGF-induced cell proliferation in MET mutant SCLC cells. WT and MET mutant cells were treated for 72 h with HGF, PHA-665752 or HGF+PHA-665752. Cell number was assessed by the trypan blue exclusion method. (**B**) The PHA-665752 inhibits HGF-induced colony formation in MET mutant SCLC cells. The SCLC cells were cultured in medium containing 0.3% agar (as described in Material and Methods) in the presence of HGF and PHA for 21 days. (**C**) The PHA-665752 decreases cell invasion in MET mutant SCLC cells. Cells were grown in serum-free media and seeded into the inserts of 24-well (8 *μ*m pore size) CHEMICON Invasion Chamber in the presence of PHA-665752 (0.5 *μ*M). Inserts were placed into Falcon companion plates containing 10% FBS and 40 ng ml^−1^ HGF and incubated for 24 h. The number of invading cells on the underside of the membrane was determined using crystal violet staining.

We then assayed the effects of HGF and PHA-665752 or the combination on cell viability in selected cell lines. PHA-665752 treatment inhibited growth in the H1993 *MET* amplified cell line. There was increased proliferation upon HGF treatment in both mutant H69 and H69AR cell lines (30–60% increase) and this effect was prevented by PHA-665752 at 0.5 *μ*M. Neither the proliferative effect nor the inhibitory effects were observed in the *MET* wild-type H524, H345 and H187 cell lines (Figure 2A).

### PHA-665752 inhibits colony formation and invasiveness in *MET* mutant SCLC cells

Exposure of H69 to HGF (40 ng ml^−1^) doubled colony formation in soft agar. Consistent with the observation that H69 is dependent on MET signalling, PHA-665752 at 0.5 *μ*M reduced colony formation in H69 cells (Figure 2B), both in non-stimulated and HGF-stimulated conditions (*P*<0.05). This effect was also observed in H69AR and correlated with inhibition of MET phosphorylation (data not shown). In contrast, these effects were not observed in the H187, H345 and H524 wild-type cell line (Figure 2B). These experiments are consistent with the inhibition of clonogenic growth as associated with inhibition of the MET phosphorylation in *MET* mutant cell lines but not in wild-type cells.

To analyse the effect of MET inhibition on invasive capacity of SCLC cell lines we then performed a CHEMICON cell invasion assay. We used HGF as a chemoattractant into the well containing culture medium. Treatment with PHA-665752 0.5 *μ*M decreased invasion of H69 by 52%, H69AR by 50% and H1993 by 47% (*P*<0.05). In contrast, for the MET wild-type H187, H345 and H524 invasion was not influenced by treatment with PHA-665752 (Figure 2C). These results support a role for MET inhibition in abrogating MET-dependent cells’ invasive capacity.

### MET and p-MET expression show different patterns in human SCLC

As MET phosphorylation seems to be a good indicator of the activation of the MET pathway, we explored the implication of the expression of this marker in tumour samples. We sought to evaluate the pattern and prevalence of total MET and p-MET expression in a series of 77 human SCLC samples. The vast majority of the assayed specimens were from primary tumours (*n*=72). Four were from regional lymph nodes and one from a distant site. Patients’ characteristics are listed in Table 1.

The MET expression in SCLC samples was consistent using two different primary antibodies (3D4 and SP44) (*P*=0.009, *R*^2^=0.85). The MET phosphorylation was assessed using two antibodies: Y1349 (docking site of MET) and Y1234/35 (autophosphorylation site of MET). Results in Supplementary Figure 2 show a high correlation (*P*<0.001, *R*^2^=0.73) between both antibodies. Strong agreement was detected in MET and p-MET scores between two observers (MET *P*<0.001, *R*^2^=0.852 and p-MET *P*=0.001, *R*^2^=0.642) and mean of values were considered for statistical analysis. The ROC was used to determine the optimal cutoff points for MET and p-MET overexpression, which were calculated at Hscore of 120 and Hscore of 5, respectively. At this value, the sensitivity of the test for MET was 57.1%, with a specificity of 62.7%, and the sensitivity for p-MET was 46.3%, with a specificity of 85.7% (Figure 4).

A total of 58 (75.3%) specimens showed any grade of MET expression and 42 (54.5%) were considered as tumours with MET overexpression. The MET staining was consistently observed in the membrane of tumour cells, and the expression was diffuse in the tumour. Differences in intensity of MET expression were occasionally detected in the same tumour.

**Figure 3:**
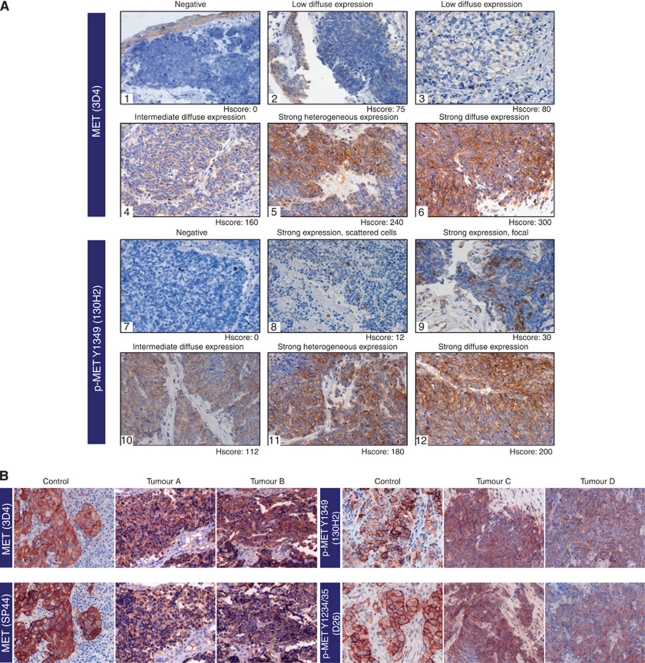
(**A**) MET and p-MET expression patterns in SCLC. (1–6) Different patterns of total MET expression in human SCLC (normal epithelia and tumour), assayed by immunohistochemistry. (7–12) Different patterns of p-MET (Y1349) expression in human SCLC. Negative expression observed in tumour 7. Strong expression of p-MET in scattered cells (tumour 8), focal (tumour 9) or diffuse (tumour 12). Intermediate or combined strong and intermediate expression patterns were observed in tumours 10 and 11, respectively. Diffuse: staining of a majority or all tumour cells; heterogeneous: presence of positive tumour areas while others negative. (**B**) Immunohistochemistry for MET and p-MET in consecutive tissue sections from FFPE lung cancer specimens. A NSCLC (control) sample with well-known levels of MET and p-MET was used in 3D4 and SP44 (MET) and 130H2 and D26 (p-MET) assays. In SCLC samples, same intensity and pattern of staining were observed for MET expression (tumours A and B) and for p-MET (tumours C and D).

Thirty-three (42.9%) cases showed p-MET overexpression following the ROC criteria. All p-MET positive cases expressed the total form of the receptor. The pattern of p-MET staining was membranous and varied from a few cells showing positivity to diffuse staining in other tumours (Figure 3A).

Sixty-eight cases had also histologically normal bronchial epithelium representation in the section. Total MET staining was faintly detected in non-tumour epithelial cells in 67 (98%) specimens and p-MET staining in 16 (24%) (Figure 3A).

We next evaluated the association of clinical variables with MET and p-MET status. The only significant association (*P*=0.048) we found was between p-MET positivity and extensive stage (Table 2).

### MET activation is associated with decreased survival in MET-positive SCLC

To provide data regarding the prognostic impact of MET expression and activation in human SCLC, we performed survival analysis of our series of patients stratified by the status of the markers. We first evaluated the association of clinical variables with overall survival (OS). As expected, patients with limited stage had a significantly better survival than patients with advanced disease (*P*<0.001). Performance status and gender were not significantly linked to survival in our series in the univariate analysis.

**Figure 4:**
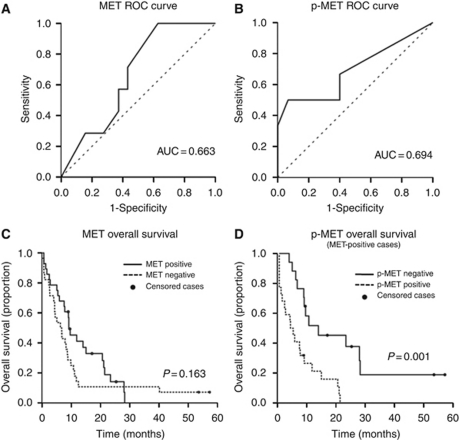
MET and p-MET overexpression threshold and prognostic significance in the cohort of SCLC patients. (**A**, **B**) The ROC was used to determine the optimal cutoff point for MET and p-MET expression. The MET and p-MET thresholds according to ROC data were 120 and 5, respectively. At these Hscore values, the sensitivity of the MET test was 57.1%, with a specificity of 62.7% for MET; and the sensitivity of p-MET assay was 46.3%, with a specificity of 85.7%. These scores were used to define overexpression. (**C**) Association between MET expression and overall survival. (**D**) Association between p-MET expression and overall survival. *P*-values were calculated using the log-rank test and survival curves by Kaplan–Meier analysis.

MET expression in SCLC patients was not significantly associated with prognosis (median OS: 270 days in MET overexpression; median OS: 203 days in low/negative MET expression) (*P*=0.163) (Figure 4).

In contrast, p-MET overexpression in SCLC was significantly associated with a different clinical outcome (*P*=0.001). Those patients with p-MET overexpression had worse prognosis (OS: 132 days) compared with p-MET negative/low expression cases (OS: 287 days). Moreover, an inverse significant correlation was detected between time of survival and levels of p-MET expression (*P*=0.022). The range of results for Hscore for p-MET was from 0 to 150. There was a trend towards an association between higher score and worse outcome (*P*=0.091). Phospho-MET was assayed with Y1349 in the whole series (Figure 4). Due to lack of sufficient tissue for additional testing in many cases, Y1234/35 was assayed only in 30 specimens. There was a good correlation between the staining with the two antibodies, however, a survival analysis with Y1234/35 was not performed due to reduced number of cases. We performed an additional analysis selecting the 10 cases with highest p-MET expression compared with the rest and observed that median survival was extremely poor in this highest expression group (80.5 days *vs* 270 days) (Supplementary Figure 3). This suggests the possibility that the level of p-MET expression may influence patient outcome, albeit much larger series would be needed to study this hypothesis.

Finally, in a multivariate analysis including disease stage (the only clinical significant factor in univariate analysis) (Table 3) and p-MET status, both parameters remained as independent variables associated with OS (*P*<0.05).

### *MET* mutations in SCLC

To explore whether *MET* mutations and p-MET staining were related, we analysed the previously described *MET* mutations in SCLC samples and cell lines: R988C, T1010I and E168C in our specimens. The 37, 20 and 33 samples with enough remaining material were evaluated for each mutation respectively. No mutations were found in these codons, thus precluding the analysis of their potential relationship with p-MET. Of note, however, *MET* mutations have been described in human SCLC and larger series may allow to analyse this correlation.

## Discussion

Here we demonstrate that MET inhibition, using PHA-665752, is able to counteract HGF-induced effects in *MET* mutant SCLC cells but not in *MET* wild-type SCLC cells. The magnitude of the effects was greater in terms of reduced clonogenicity and invasiveness when the inhibitor was used at relatively low concentrations, consistent with those needed to inhibit the target. Furthermore, we provide novel evidence for the activation of MET in about 40% of patients with SCLC and a link with poor outcome, independent of disease stage.

An intriguing finding was that although H69 and H69AR harbour a *MET* mutation, basal levels of phosphorylation were low in both cell lines and only when stimulated with HGF this phosphorylation increased significantly as well as the oncogenic phenotype. Previous studies have suggested that stimulation with HGF is necessary to display the oncogenic properties of cells carrying *MET* mutations, which is in line with our findings and could be considered an example of oncogene expedience (Michieli *et al*, 1999; Comoglio *et al*, 2008). This term has been proposed by Professor Comoglio's group and supports the concept of activation of MET as a secondary event that exacerbates the malignant properties of already transformed cells. In our model *MET* mutation is not acting as a driver, which may define oncogene addiction but rather as a secondary phenomenon that influences biology of the mutant cell. Our data show that cancer cells with mutant *MET* (not the wild type) and MET pathway activation via exogenously added HGF have an increased proliferation, invasiveness and colony formation capacity. Some of these effects have been previously described for SCLC cell lines (Maulik *et al*, 2002; Ma *et al*, 2003; Jagadeeswaran *et al*, 2007). Specific inhibition of MET phosphorylation with small molecule inhibitors has demonstrated the capacity of counteracting the HGF induced effects in SCLC and other tumour models (Christensen *et al*, 2003; Ma *et al*, 2005; Zou *et al*, 2007). Here we have not only confirmed these observations but also extended them by providing direct evidence for anti-oncogenic properties of a small molecule inhibitor, PHA-665752, in *MET* mutant SCLC cell lines, independently of their sensitivity/resistance to chemotherapy.

Our work demonstrates that the R988C mutation in our cell line model does not act as a driver mimicking the oncogene addiction phenomenon observed in MET amplified cases (Smolen *et al*, 2006; Lutterbach *et al*, 2007). This is in line with a recent work that demonstrates lack of functionality of these mutations as oncogenes and are thus considered passenger mutations (Tyner *et al*, 2010). In our work, the R988C mutation sensitises cells to the effect of HGF and to the effect of the MET inhibitor. This stresses the importance of HGF paracrine/endocrine function in *MET* mutant tumours that is involved in angiogenesis, growth, migration and invasion (Shojaei *et al*, 2010) and the potential role of MET inhibitors in cases with *MET* mutations. There are two studies that report the association between increased HGF serum levels in patients with SCLC and more advanced disease and prognosis (Takigawa *et al*, 1997; Bharti *et al*, 2004). However, to our knowledge no study has evaluated the correlation of HGF serum levels and MET expression in SCLC specimens.

There are many studies evaluating the expression of MET but a limited number with p-MET in human tumours and none of them have focused in SCLC (Kuniyasu *et al*, 1992). Our work demonstrates that MET was expressed in >70% of human SCLC, but that p-MET is expressed in 40% of cases. As expected, MET was also expressed in a high proportion of non-malignant epithelial cells adjacent to malignant tissue. MET expression has been previously reported in non-tumour lung tissue and, in a similar manner of what we report here, specifically a weak expression in the basal aspect of the bronchial epithelium (Olivero *et al*, 1996; Chen *et al*, 2006; Ma *et al*, 2008). In addition to its physiological role in lung epithelial homeostasis, the MET pathway appears to be critical in the repair of lung damage induced by chronic tobacco exposure (Chen *et al*, 2006). It is tempting to speculate from this observation that cells with persistent MET activation in the context of tobacco use might be more susceptible to malignant transformation by posterior oncogenic mutations (Pleasance *et al*, 2009).

From a clinical perspective, the most relevant finding was the prognostic role of p-MET expression in SCLC. As MET phosphorylation is a surrogate marker of receptor activation, this finding is consistent with an adverse role of activated MET receptor in SCLC. This is in agreement with previous reports on the role of p-MET in other tumour types. The prognostic value of p-MET was independent of disease stage, suggesting that it might have a role in the adverse clinical behaviour of patients with this marker. The antibody we used for p-MET expression analysis Y1349 is the epitope of the docking site of MET receptor. Upon HGF binding, MET receptor is activated through dimerisation and phosphorylation of Tyr1234/35 on the catalytic domain. Subsequently phosphorylation of docking sites Tyr1349/56 occurs with recruitment of downstream molecules in our cell line models and has been reported previously (Ponzetto *et al*, 1994). The connection between both phosphorylation processes and the equivalent results obtained with the Y1234/35 antibody support the role of MET phosphorylation as a negative prognostic marker in this disease.

Secondly and in contrast, total (activation independent) MET expression was not associated with prognosis in our series. In fact, in the literature, the prognostic role of total MET in human cancer is controversial. Albeit many studies point to an adverse prognostic role (Birchmeier *et al*, 2003; Go *et al*, 2010; Kim *et al*, 2010; Liu *et al*, 2011), other point to the contrary (Furukawa *et al*, 1995; Belfiore *et al*, 1997; Nakamura *et al*, 2007; Cappuzzo *et al*, 2009; Kanteti *et al*, 2009). Further studies will be needed to solve this issue.

Heterogeneity in MET expression in whole tissue sections in other tumours has been described (Ma *et al*, 2007; Benedettini *et al*, 2010). One of the caveats of our immunohistochemical studies is that they were performed in small biopsies (usual material obtained in this disease) and therefore we could not assess heterogeneity in MET expression in SCLC.

Finally, in order to correlate our preclinical work with our clinical findings we performed mutational studies in cases with available tissue. Due to limited material we were able to study only previously described *MET* mutations in SCLC in a subset of the whole population. No mutations were found in this sample set. However, *MET* mutations have been previously described in SCLC specimens (Ma *et al*, 2003) and although probably infrequent, we believe further studies with larger number of samples are needed to definitively address this area. This is now underway on a prospective project in our institution.

In conclusion, our results add support to the view of MET as an attractive therapeutic target for a subgroup of SCLCs-harbouring *MET*-activating mutations. They also argue in favour of testing MET targeting agents preferentially in patients whose tumours display p-MET expression or *MET* mutations.

## Figures and Tables

**Table 1:** Clinical characteristics of small cell lung cancer patients

**Patients’ characteristics**	**Number**
Median age (range)	65 (41–85)
	
*Gender*	
Female	9 (12%)
Male	68 (88%)
	
*Smoking history*	
Never	1 (1%)
Former	22 (29%)
Current	54 (70%)
	
*Performance status*	
0–1	48 (62%)
2–3	13 (17%)
Unknown	16 (21%)
	
*Clinical stage*	
Limited	32 (42%)
Extensive	42 (54%)
Unknown	3 (4%)

**Table 2:** Association between MET and p-MET expression and clinical variables

	**Total MET expression**			**p-MET expression**		
	**POS**	**NEG**	***P*-value**	**POS**	**NEG**	***P*-value**
*Gender*						
Female	5	4	0.948	3	6	0.539
Male	37	31		30	38	
						
*Smoking history*						
Previous/never	16	8	0.253	8	16	0.453
Current	25	27		24	28	
						
*PS*						
0–1	29	19	0.544	21	27	0.855
2–3	8	5		5	8	
						
*Stage*						
Limited	15	17	0.381	9	23	0.048
Extensive	24	18		21	21	

Abbreviations: NEG=negative; p-MET=phosphorylated MET; POS=positive; PS=performance status.

**Table 3:** Cox multivariate model for overall survival

	**HR (95% CI)**	***P*-value**
Stage (extensive *vs* limited)	2.96 (1.58–5.54)	0.001
p-MET overexpression (no *vs* yes)	1.93 (1.07–3.48)	0.028

Abbreviations: CI=confidence interval; HR=hazard ratio.
