# Supplementary figures and images for: MET phosphorylation predicts poor outcome in small cell lung carcinoma and its inhibition blocks HGF-induced effects in MET mutant cell lines
Source: Br J Cancer. 2011 Aug 16;105(6):814–23. doi: 10.1038/bjc.2011.298 (PMC3171012; doi:10.1038/bjc.2011.298)

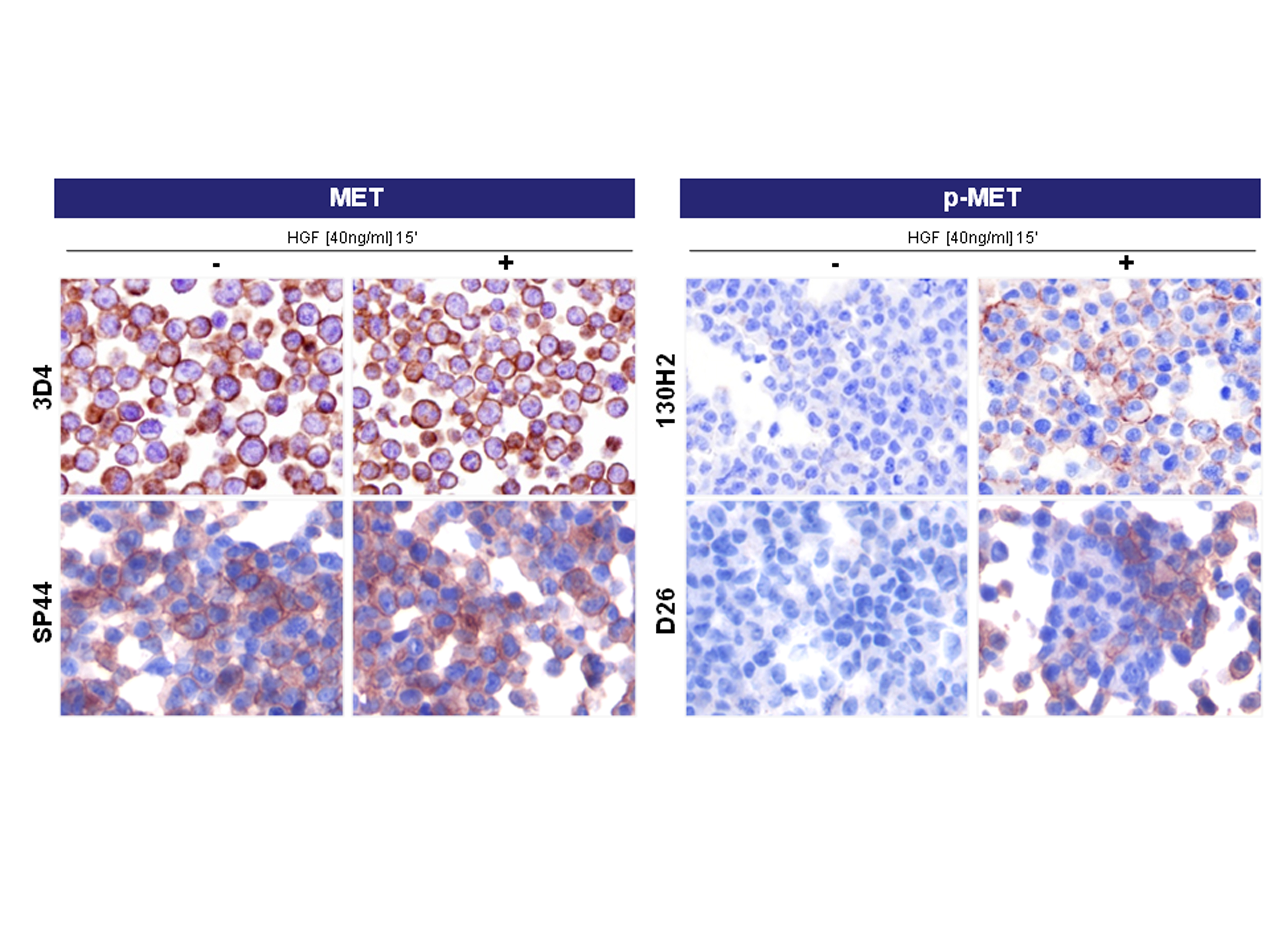

Supplement: Supplementary Figure 1 [file bjc2011298x1.tif]

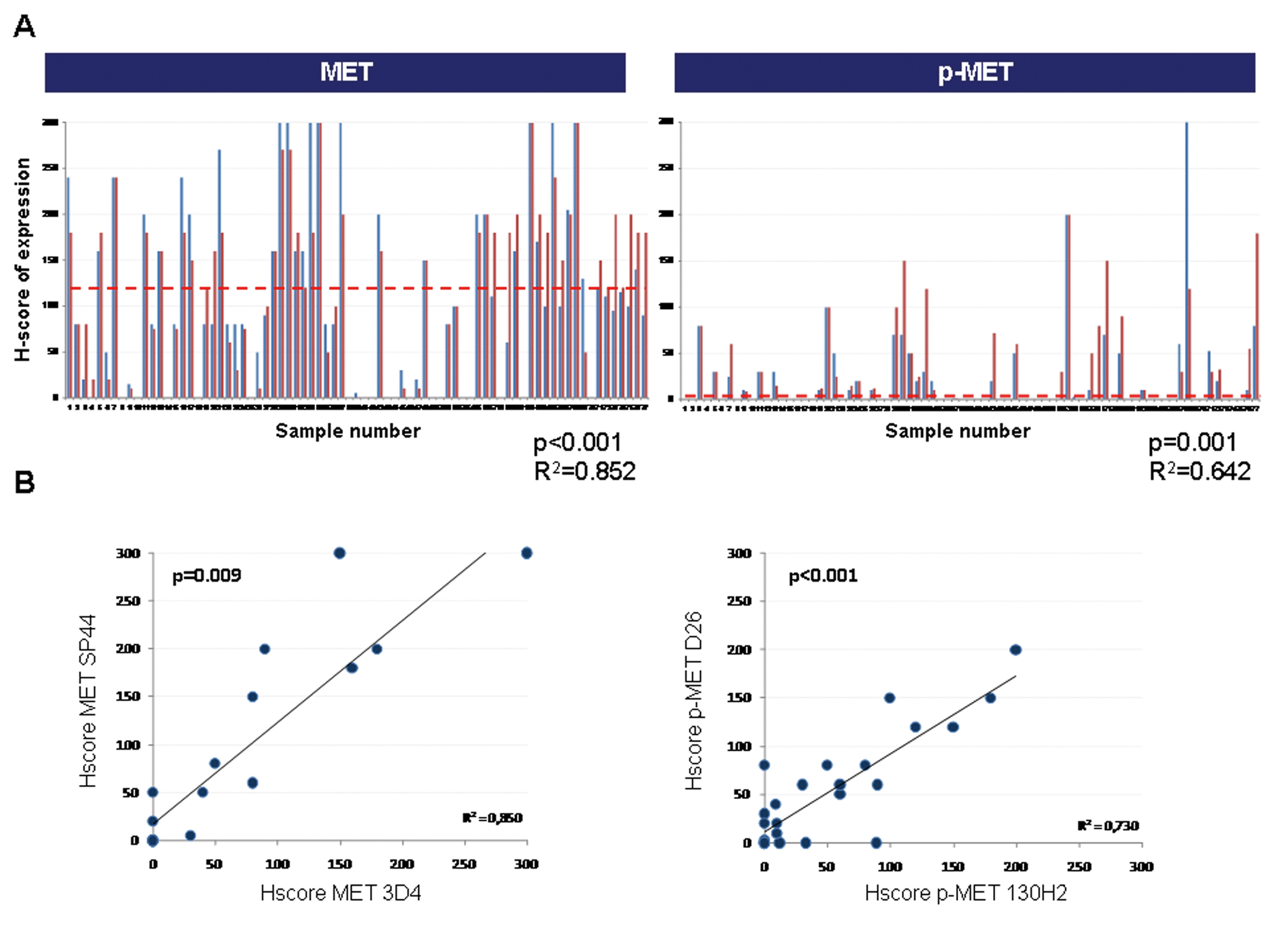

Supplement: Supplementary Figure 2 [file bjc2011298x2.tif]

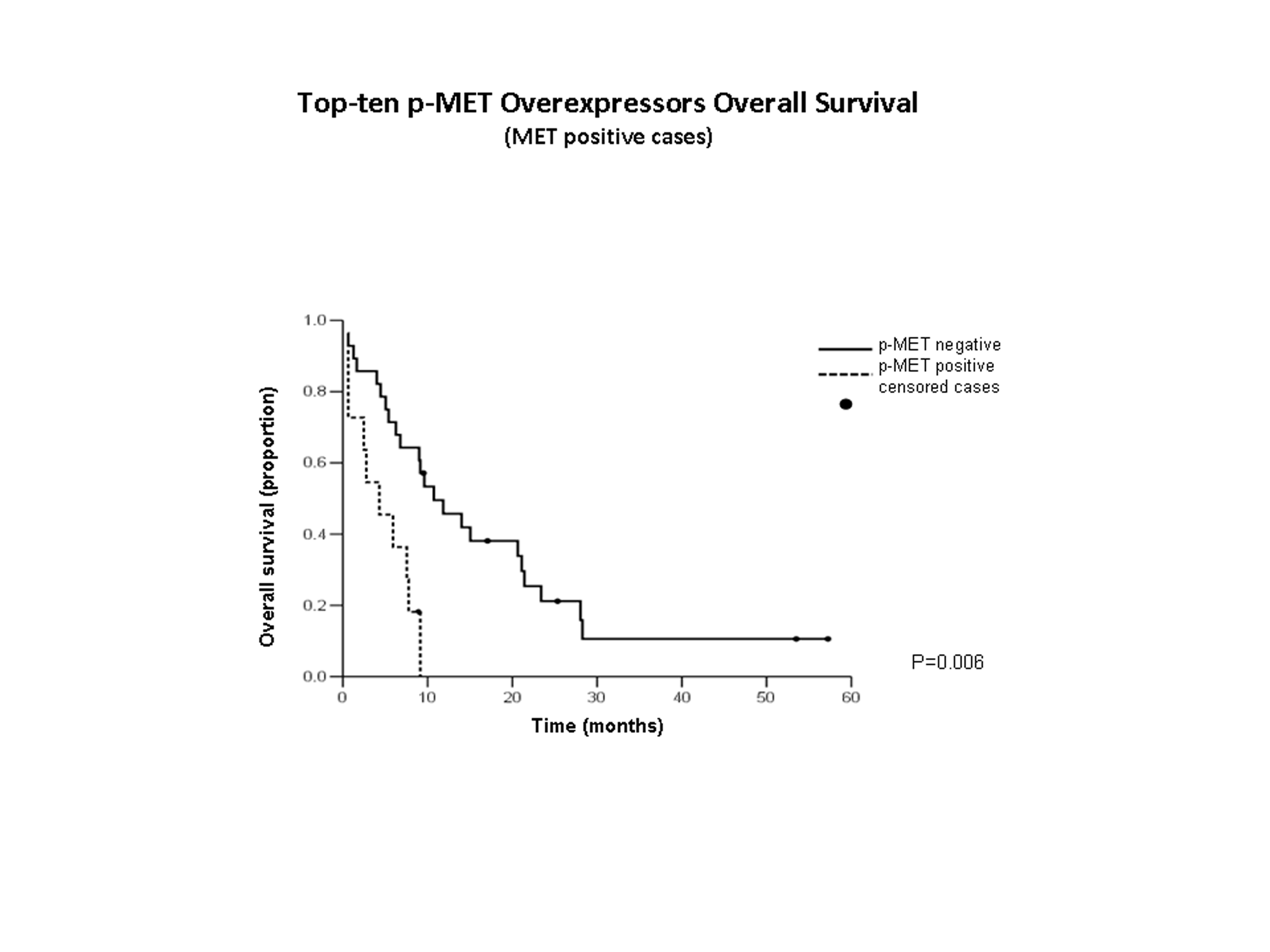

Supplement: Supplementary Figure 3 [file bjc2011298x3.tif]
