# Supplementary material for: MET phosphorylation predicts poor outcome in small cell lung carcinoma and its inhibition blocks HGF-induced effects in MET mutant cell lines
Source: Br J Cancer. 2011 Aug 16;105(6):814–23. doi: 10.1038/bjc.2011.298 (PMC3171012; doi:10.1038/bjc.2011.298)
Supplement: Supplementary Figure Legends [file bjc2011298x4.doc]

**Supplementary Figure legends**

**Supplementary Figure 1.** Validation of specificity and sensitivity of the MET and p-MET IHC assays. Immunocytochemistry in FFPE H69 cells, at basal and HGF-stimulated conditions (40mg/ml for 15 min). MET receptor, using both 3D4 and SP44 antibodies, was detected as membranous expression in basal and stimulated conditions, showing same intensity of staining. p-MET was only present at membrane in the HGF-stimulated H69, observing same pattern of expression with 130H2 and D26 antibodies. Specificity of experiments were corroborated by Western blot in whole lysates from same H69 cells. **Supplementary Figure 2.** Correlation of MET and p-MET Hscores between observers and antibodies. A. Hscores from two observers were displayed for MET and p-MET assays, showing a strong correlation in expression values (p<0.001; R^2^=0.852 for MET, and p=0.001; R^2^=0.642 for p-MET). B. Significant correlations between MET (p=0.009; R^2^=0.850) and p-MET (p<0.001; R^2^=0.730) Hscores, using two different antibodies (3D4 and SP44 for MET; 130H2 and D26 for p-MET) in a subset of SCLC samples.

**Supplementary Figure 3**. Kaplan-Meier overall survival curves for patients with the highest expression of p-MET compared to the rest.
